# Cytogenetic and interphase Fluorescence *in Situ* Hybridization studies in patients with multiple myeloma

**DOI:** 10.1186/1755-8166-7-S1-P20

**Published:** 2014-01-21

**Authors:** G Perumal, RS Chandra, P Prabu, N Indhumathi, Anil Tarigopula, Rama Mani

**Affiliations:** 1 Department of Molecular Biology and Cytogenetics, Apollo Hospitals, No. 21, Greams Lane, Off. Greams Road, Chennai-600 006, Tamilnadu, India; 2 Department of Genetics, Dr. ALM PG. Institute of Basic Medical Sciences, University of Madras, Taramani, Chennai - 600113, Tamilnadu, India

## Background

Multiple myeloma (MM) is characterized by the clonal proliferation and accumulation of malignant plasma cells in the bone marrow, monoclonal protein in the blood or urine and associated organ dysfunction. Some patients may show a slow progressive evolution from monoclonal gammopathy of undetermined significance while others may be associated with features of high clonal aggressiveness such as plasma cell leukemia or extramedullary plasmacytomas. Chromosomal abnormalities (mainly IgH translocations and trisomies) have been shown to be of prognostic significance in MM. Interphase fluorescence in situ hybridization (i-FISH), in particular, has been much more effective in identifying these trisomies/ monosomies and specific translocations.

## Materials and methods

The bone marrow aspirates were processed for conventional cytogenetic and interphase FISH analyses using three probes to detect del(13)(q14.3), t(4;14)(p16.3:q32) and del(17)(p13.1). A total of 30 newly diagnosed patients were studied between May 2012 and March 2013. The affected were mostly elderly people with median age of 55 years (range: 32 to 80 years) at the time of diagnosis. There were 21 males and 9 females.

## Results and conclusions

Chromosomal abnormalities were detected in only 7 patients because of the low proliferation rate of plasma cells and the non-availability of analyzable metaphases. On the other hand, i-FISH revealed an abnormality in 14 patients and a normal pattern for the selected probes in the remaining 16 patients. The most frequent abnormality was found to be 13 monosomy (complete/ partial) in 13 cases followed by t(4;14) seen in 4 patients. However these abnormalities were not recognized in the karyograms. None of the cases showed a p53 deletion. Further studies employing the complete FISH panel are required for better diagnosis and prognosis.

